# Work Motivation: The Roles of Individual Needs and Social Conditions

**DOI:** 10.3390/bs12020049

**Published:** 2022-02-15

**Authors:** Thuy Thi Diem Vo, Kristine Velasquez Tuliao, Chung-Wen Chen

**Affiliations:** 1 Department of Business Administration, National Taiwan University of Science and Technology, No. 43, Section 4, Keelung Road, Da’an District, Taipei City 106335, Taiwan; d10708813@mail.ntust.edu.tw (T.T.D.V.); cwchen@mail.ntust.edu.tw (C.-W.C.); 2 Graduate Institute of Human Resource Management, National Central University, No. 300, Zhongda Road, Zhongli District, Taoyuan City 320317, Taiwan

**Keywords:** work motivation, psychological needs, social conditions, self-determination theory, prosocial motivation

## Abstract

Work motivation plays a vital role in the development of organizations, as it increases employee productivity and effectiveness. To expand insights into individuals’ work motivation, the authors investigated the influence of individuals’ competence, autonomy, and social relatedness on their work motivation. Additionally, the country-level moderating factors of those individual-level associations were examined. Hierarchical linear modeling (HLM) was used to analyze data from 32,614 individuals from 25 countries, obtained from the World Values Survey (WVS). Findings showed that autonomy and social relatedness positively impacted work motivation, while competence negatively influenced work motivation. Moreover, the individual-level associations were moderated by the country-level religious affiliation, political participation, humane orientation, and in-group collectivism. Contributions, practical implications, and directions for further research were then discussed.

## 1. Introduction

Work motivation is considered an essential catalyst for the success of organizations, as it promotes employees’ effective performance. To achieve an organization’s objectives, the employer depends on the performance of their employees [1]. However, insufficiently motivated employees perform poorly despite being skillful [1,2]. Employers, therefore, need their employees to work with complete motivation rather than just showing up at their workplaces [3]. Work motivation remains a vital factor in organizational psychology, as it helps explain the causes of individual conduct in organizations [4]. Consequently, studies on the factors that encourage work motivation can contribute to the theoretical underpinnings on the roots of individual and practical social conditions that optimize individuals’ performance and wellness [5].

Several decades of research have endeavored to explain the dynamics that initiate work-related behavior. The primary factor examining this aspect is motivation, as it explains why individuals do what they do [6]. The basic psychological needs have represented a vital rationalization of individual differences in work motivation. Psychological needs are considered natural psychological nutrients and humans’ inner resources. They have a close relationship with individual conduct and have a strong explicit meaning for work performance [7,8]. Different needs are essential drivers of individual functioning due to the satisfaction derived from dealing with them [9]. In addition to individual-level antecedents, the social context has also been regarded to have implications for work motivation. Social exchange and interaction among individuals accentuate the importance of work motivation as something to be studied with consideration of contextual factors [10].

Significant contributions have been made to the socio-psychological perspective of work motivation (Table 1). However, current literature shows three deficiencies. First, over 150 papers utilize the key approaches of psychological needs to justify motivational processes in the workplace [11], which justifies the vital role of psychological needs in interpreting individual work motivation. The association between psychological needs and work motivation has often been implicitly assumed; however, the influence of psychological needs on work motivation has been inadequately tested [8]. The verification of the extent and the direction of influence will provide a better understanding of, and offer distinct implications for, the facilitation of work motivation. In examining the influence of psychological needs on work motivation, this paper mainly focuses on the intrinsic aspect of motivation. The study of Alzahrani et al. (2018) [12] argued that although intrinsic motivation is more efficient than extrinsic motivation, researchers have mostly neglected it.

Second, there is no study examining the country-level moderating effects of social conditions and national cultures on individual relationships between psychological needs and work motivation. Pinder (2014) [20] argued that contextual practices could influence variables at the individual level. Culture is a crucial factor influencing motivation [15,16,17,18]. Researchers (e.g., [19]) have further suggested that both the proximal social situations (e.g., workgroup) and the distal social situations (e.g., cultural values) in which humans operate influence their need for satisfaction and their motivation type. Intrinsic motivation interacts with prosocial motivation in judging work performance [21]. By including the social conditions in the framework, prosocial motivation is considered. Prosocial motivation refers to the desire to help and promote the welfare of others [22,23]. The study of Shao et al. (2019) [24] proposed that prosocial motivation promotes employee engagement in particular organizational tasks. Researchers often consider prosocial motivation as a pattern of intrinsic motivation [23]. This implies that when intrinsic motivation is investigated, prosocial motivation should be examined together to obtain a comprehensive understanding.

Third, there are few studies using a considerable number of cross-national samples to investigate factors influencing work motivation. A cross-cultural analysis makes the findings more objective by minimizing individual bias towards any particular culture. Therefore, the examination of the study is crucial to expanding insights on the influence of social situations on the individual associations between psychological needs and work motivation.

## 2. Literature Review and Hypothesis Development

### 2.1. Work Motivation: A Conceptual Background

Work motivation is considered “a set of energetic forces that originate both within as well as beyond an individual’s being, to initiate work-related behavior, and to determine its form direction intensity and duration” [20]. Nicolescu and Verboncu (2008) [25] argued that work motivation contributes directly and indirectly to employees’ performance. Additionally, research (e.g., [26]) has postulated that work motivation could be seen as a source of positive energy that leads to employees’ self-recognition and self-fulfillment. Therefore, work motivation is an antecedent of the self-actualization of individuals and the achievement of organizations.

Literature has identified several models of work motivation. One of the primary models is Maslow’s (1954) [27] need hierarchy theory, which proposes that humans fulfill a set of needs, including physiological, safety and security, belongingness, esteem, and self-actualization. Additionally, Herzberg’s (1966) [28] motivation-hygiene theory proposed that work motivation is mainly influenced by the job’s intrinsic challenge and provision of opportunities for recognition and reinforcement. More contemporary models also emerged. For instance, the study of Nicolescu and Verboncu (2008) [25] has categorized the types of motivation into four pairs, including positive-negative, intrinsic-extrinsic, cognitive-affective, and economic-moral spiritual. Additionally, Ryan and Deci [29] focused on intrinsic motivation and extrinsic motivation.

With the existence of numerous factors that relate to work motivation, this paper mainly focuses on intrinsic motivation. Previous research found that emotional intelligence and interpersonal relationship quality predict individuals’ intrinsic motivation [14]. Additionally, the study of Lin (2020) [13] argued that personal factors, including age, gender, educational level, living setting, health status, and family support, impact people’s intrinsic motivation. To understand more about intrinsic motivation, the authors examined individuals’ psychological needs. Fulfillment of the basic needs is related to wellness and effective performance [7]. Since intrinsic motivation results in high-quality creativity, recognizing the factors influencing intrinsic motivation is important [5].

Although a significant number of important contributions have been made regarding intrinsic motivation, self-determination theory is of particular significance for this study. Self-determination theory (SDT) postulates that all humans possess a variety of basic psychological needs. One of the primary crucial needs is the need for competence [30,31], which makes individuals feel confident and effective in their actions. Additionally, the need for autonomy [32] is one of the important psychological needs, which makes people satisfied with optimal wellness and good performance obtained as a result of their own decisions. Moreover, SDT proposed the crucial importance of interpersonal relationships and how social forces can influence thoughts, emotions, and behaviors [33]. This means that the psychological need for social relatedness [34] also plays a significant role in human’s psychological traits. Individuals need to be cared for by others and care for others to perceive belongingness. The need for relatedness can motivate people to behave more socially [35].

Prior research (e.g., [36]) has explored self-determination theory and related theories as approaches to work motivation and organizational behavior. The study of Van den Broeck et al. (2010) [37] emphasized grasping autonomy, competence, and relatedness at workplaces. This paper contributes to the exhaustive understanding of intrinsic work motivation influenced by further examining the impact of these three factors on work motivation as well as the moderating effects of social contexts.

### 2.2. Main Effect

#### 2.2.1. Individuals’ Competence and Work Motivation

Competence is “the collective learning in the organization, especially how to coordinate diverse production skills and integrate multiple streams of technologies” [38]. The study of Hernández-March et al. (2009) [39] argued that a stronger competence was commonly found in university graduates rather than those without higher education. Competence has been considered a significant factor of work motivation that enhances productivity and profits. Harter’s (1983) [40] model of motivation proposed that competence enhances motivation because competence promotes flexibility for individuals [41]. Likewise, Patall et al. (2014) [42] indirectly argued that competence positively affects work motivation. Individuals become more engaged in activities that demonstrate their competence [6]. When people perceive that they are competent enough to attain goals, they generally feel confident and concentrate their efforts on achieving their objectives as soon as possible for their self-fulfillment.

**Hypothesis** **1** **(H1).** *Individuals’ competence positively relates to their work motivation.*

#### 2.2.2. Individuals’ Autonomy and Work Motivation

Autonomy is viewed as “self-determination, self-rule, liberty of rights, freedom of will and being one’s own person” [43]. Reeve (2006) [44] argued that autonomy is a primary theoretical approach in the study of human motivation and emotion. Autonomy denotes that certain conduct is performed with a sense of willingness [30]. Several researchers (e.g., [45]) investigated the positive relationship between individuals’ autonomy and work motivation. When humans are involved in actions because of their interest, they fully perform those activities volitionally [36]. Dickinson (1995) [46] also proposed that autonomous individuals are more highly motivated, and autonomy breeds more effective outcomes. Moreover, when individuals have a right to make their own decisions, they tend to be more considerate and responsible for those decisions, as they need to take accountability for their actions. Bandura (1991) [47] has argued that humans’ ability to reflect, react, and direct their actions motivates them for future purposes. Therefore, autonomy motivates individuals to work harder and overcome difficulties to achieve their objectives.

**Hypothesis** **2** **(H2).** *Individuals’ autonomy positively relates to their work motivation.*

#### 2.2.3. Individuals’ Social Relatedness and Work Motivation

The psychological need for social relatedness occurs when an individual has a sense of being secure, related to, or understood by others in the social environment [48]. The relatedness need is fulfilled when humans experience the feeling of close relationships with others [49]. Researchers (e.g., [34]) have postulated that the need for relatedness reflects humans’ natural tendency to feel associated with others, such as being a member of any social groups, or to love and care as well as be loved and cared for. Prior studies have shown that social relatedness strongly impacts motivation [50,51,52]. Social relatedness offers people many opportunities to communicate with others, making them more motivated at the workplace, aligning them with the group’s shared objectives. Marks (1974) [53] suggested that social relatedness encourages individuals to focus on community welfare as a reference for their behavior, resulting in enhanced work motivation. Moreover, when individuals feel that they relate to and are cared for by others, their motivation can be maximized since their relatedness need is fulfilled [54]. Therefore, establishing close relationships with others plays a vital role in promoting human motivation [55]. When people perceive that they are cared for and loved by others, they tend to create positive outcomes for common benefits to deserve the kindness received, thereby motivating them to work harder.

**Hypothesis** **3** **(H3).** *Individuals’ social relatedness positively relates to their work motivation.*

Aside from exploring the influence of psychological needs on work motivation, this paper also considers country-level factors. Previous research (e.g., [56]) has examined the influence of social institutions and national cultures on work motivation. However, the moderating effects of country-level factors have to be investigated, given the contextual impacts on individual needs, attitudes, and behavior. Although social conditions provide the most common interpretation for nation-level variance in individual work behaviors [57], few cross-national studies examine social conditions and individual work behaviors [56]. Hence, this paper investigates the moderating effects, including religious affiliation, political participation, humane orientation, and in-group collectivism, on the psychological needs-work motivation association.

A notable theory to explain the importance of contextual factors in work motivation that is customarily linked with SDT is the concept of prosocial motivation. Prosocial motivation suggests that individuals have the desire to expend efforts in safeguarding and promoting others’ well-being [58,59]. It is proposed that prosocial motivation strengthens endurance, performance, and productivity, as well as generates creativity that encourages individuals to develop valuable and novel ideas [21,60]. Prosocial motivation is found to interact with intrinsic motivation in influencing positive work outcomes [21,61]. However, there are few studies examining the effects of prosocial motivation on work motivation [62].

Utilizing the concept of prosocial motivation and examining it on a country-level, this paper suggests that prosocial factors promote basic psychological needs satisfaction that reinforces motivational processes at work. Therefore, prosocial behaviors and values may enhance the positive impact of individuals’ basic psychological needs, including competence, autonomy, and social relatedness, on work motivation. 

### 2.3. Moderating Effects

#### 2.3.1. Religious Affiliation

Religions manifest values that are usually employed as grounds to investigate what is right and wrong [63]. Religious affiliation is considered prosocial because it satisfies the need for belongingness and upholds collective well-being through gatherings to worship, seek assistance, and offer comfort within religious communities. Hence, religious affiliation promotes the satisfaction of individuals’ psychological needs, which directs motivation at work and life in general. Research (e.g., [64]) has argued that religious affiliation is an essential motivational component given its impact on psychological processes. The study of Simon and Primavera (1972) [65] investigated the relationship between religious affiliation and work motivation. To humans characterized by competence, autonomy, and social relatedness, attachment to religious principles increases their motivation to accomplish organizational goals. Religious membership will increase the influence of psychological needs on work motivation. The tendency of individuals affiliated with any religion to be demotivated is lower compared to those who are not. Individuals with religious affiliations also tend to work harder as the virtue of hard work is aligned with religious principles. Accordingly, religious affiliation may enhance the positive association between individuals’ psychological needs and work motivation.

#### 2.3.2. Political Participation

Political participation, indicated by people’s voting habits, plays a crucial role in ensuring citizens’ well-being and security [66]. Political participation encourages shared beliefs and collective goals among individuals [67]. The communication and interaction among people help them grasp the government’s developmental strategies, motivating them to work harder. Political participation is a collective pursuit that makes societal members feel more confident, socially related, and motivated at work to achieve communal targets. Increased political participation reinforces effective public policy to enhance its members’ welfare, congruent with the perspectives of prosocial motivation. The prosocial values and behaviors derived from political participation satisfy human needs and interact positively with intrinsic motivation. Therefore, political participation may strengthen the positive influence of individuals’ competence, autonomy, and social relatedness on work motivation. Conversely, poor political participation is perceived as a separation from the society that may lead to demotivation. In a society with poor political participation, an individualistic mentality is encouraged, thereby decreasing the desire to pursue cooperative endeavors.

#### 2.3.3. Humane Orientation

GLOBE characterizes humane orientation as “the degree to which an organization or society encourages and rewards individuals for being fair, altruistic, generous, caring, and kind to others” [68]. Research (e.g., [69,70]) has argued that a high humane orientation encourages members to develop a strong sense of belonging, commit to fair treatment, and manifest benevolence. The desire to help others or enhance others’ well-being indicates prosocial values and behaviors [71,72]. Since humane orientation is correlated with philanthropy and promotes good relations, this cultural value may enhance work motivation. Fairness, which is derived from a humane-oriented society, is one of the most vital influences on work motivation [1]. Moreover, altruism, promoted by humane-oriented societies, encourages individuals to sacrifice individual interests for shared benefits. Altruism then encourages attachment to others’ welfare and increases resources needed for prosocial behaviors such as work [73,74]. Members of humane-oriented countries view work in a positive light—it is an opportunity for them to perform altruistic behaviors and engage in collective actions. Therefore, people are more likely to work harder for common interests in humane-oriented societies. In such conditions, individuals with competence, autonomy, and social relatedness will be more motivated to work. By contrast, a less humane-oriented society gives prominence to material wealth and personal enjoyment [75]. Although this may be perceived as a positive influence on the association between psychological needs and work motivation, such an individualistic mindset works against the prosocial factors that further motivate individuals.

#### 2.3.4. In-Group Collectivism

House et al. (2004) [68] defined in-group collectivism as “the degree to which individuals express pride, loyalty, and cohesiveness in their organizations or families”. Collectivistic cultures indicate the need for individuals to rely on group membership for identification [76]. High collectivism enhances equity, solidarity, loyalty, and encouragement [77,78]. Humans living in a collectivist culture are interdependent and recognize their responsibilities towards each other [79]. In-group collectivism transfers the concepts of social engagement, interdependence with others, and care for the group over the self (e.g., [79,80,81], thereby motivating individuals to work harder for the common interests. Oyserman et al. (2002) [82] have further argued that individualistic values encourage an independent personality, whereas collectivistic values form an interdependent one. Therefore, in-group collectivism is a prosocial value that emphasizes the importance of reciprocal relationships and encourages people to work harder to benefit the group. By contrast, low collectivism promotes individual interests and personal well-being while neglecting the value of having strong relations with others [70]. Considering that in-group collectivism promotes individuals’ prosocial behaviors of individuals, people who are competent, autonomous, and socially related to collective societies are less likely to be demotivated at the workplace. Consequently, in-group collectivism may intensify the positive influence of individuals’ competence, autonomy, and social relatedness on their work motivation.

**Hypothesis** **4** **(H4).** *(a–d): The positive relationship between individuals’ competence and their work motivation is enhanced as religious affiliation (a), political participation (b), humane orientation (c), and in-group collectivism (d) increase.*

**Hypothesis** **5** **(H5).** *(a–d): The positive relationship between individuals’ autonomy and their work motivation is enhanced as religious affiliation (a), political participation (b), humane orientation (c), and in-group collectivism (d) increase.*

**Hypothesis** **6** **(H6).** *(a–d): The positive relationship between individuals’ social relatedness and their work motivation is enhanced as religious affiliation (a), political participation (b), humane orientation (c), and in-group collectivism (d) increase.*

## 3. Methods

### 3.1. Sample

The data came from the seventh wave (2017–2021) of the World Values Survey (WVS) [83], which examines humans’ beliefs and values. This survey is performed every five years to explore changes in people’s values and perceptions. Face-to-face interviews, or phone interviews for remote areas, were conducted by local organizations. Almost 90 percent of the world’s population is represented in the WVS. At least 1000 individuals were selected as respondents to exhibit each nation’s population. Further information regarding the WVS can be reached at the WVS website (http://www.worldvaluessurvey.org, accessed on 14 October 2021).

The samples of this study were based on the availability of national-level data for the moderators and individual-level data for the measures of independent and dependent variables. Respondents without answers on the individual measures and corresponding country-level data were excluded from the analysis. The final data included 32,614 respondents in 25 countries aged 18 and above. The 25 countries included Argentina, Australia, Brazil, China, Colombia, Ecuador, Egypt, Germany, Greece, Guatemala, Hong Kong, Indonesia, Iran, Japan, Kazakhstan, Malaysia, Mexico, New Zealand, Philippines, Russia, South Korea, Taiwan, Thailand, Turkey, and the USA.

### 3.2. Dependent Variable

Consistent with previous researchers (e.g., [84]), the authors used four items to gauge individual work motivation, namely “Indicate how important work is in your life”, “People who do not work turn lazy”, “Work is a duty towards society”, and “Work should always come first, even if it means less spare”. The first item was measured on a scale from 1 to 4, in which lower scores indicate a higher level of work importance. The other three items were gauged on a scale from 1 to 5 (1 indicating strongly agree and 5 indicating strongly disagree). The scores for each item were reverse coded, and the mean scores were computed so that higher scores indicate greater work motivation.

### 3.3. Independent Variables

The independent variables of this study include individuals’ competence, autonomy, and social relatedness. First, people’s competence was measured by the item “What is the highest educational level that you attained” on a scale from 0 to 8, in which higher scores indicate a higher level of educational attainment. The authors used the item to gauge individual competence, as a capacity for learning is highlighted in the examination of competence [39]. Second, a scale from 1 to 10 was utilized to measure the item “How much freedom of choice and control”, which represented individual autonomy (1 indicating no choice at all and 10 indicating a great deal of choice). The authors used the item to gauge people’s autonomy as this item indicates the degree to which individual can make their own decisions. Finally, the individual’s social relatedness was gauged by twelve items, representing twelve types of organizations where individuals are active/inactive members or do not belong. The twelve items were measured on a scale from 0 to 2 (0 indicating do not belong, 1 indicating inactive member, and 2 indicating active member). The mean score of the twelve items represents the individual’s social relatedness. The membership in organizations represents social relatedness, as this indicates the reciprocal relationship between the individual and the organization through their mutual rights, responsibilities, and obligations towards each other [85].

### 3.4. Moderators

The four country-level moderators in this study were religious affiliation, political participation, humane orientation, and in-group collectivism. Similar to prior research (e.g., [86]), the authors used the percentage of the country’s population with religious affiliation obtained from Pew Research Center 2015 [87]. Secondly, the index of voter turnout collected from the International Institute for Democracy and Electoral Assistance [88] was utilized to gauge political participation. Voting habits are an indicator of an individual’s presence in their country’s life, and a nation with a high index of voter turnout illustrates its substantial degree of political participation [89]. Finally, two cultural values, including humane orientation and in-group collectivism, were obtained from the GLOBE study [68]. The authors used scores on cultural practices as the moderators for this study because they indicate the actual behaviors as “the way things are done in this culture” [68].

### 3.5. Control Variables

Several individual-level and country-level elements related to the dependent variable were considered control variables. The effects of gender, marital status, age, and income level were accounted for, as these four variables are basic personal factors that may impact individual’s motivation [90]. Gender (1 indicating male and 0 indicating female) and marital status (1 indicating married and 0 indicating other status) were dummy coded. Moreover, age was measured in years, while income level was gauged using a scale from 1 representing the lowest group to 10 representing the highest group. Along with the above individual-level controls, education and family strength were treated as country-level control variables. Education and family are primary institutions that shape individuals’ motivation [91,92]. Similar to prior researchers (e.g., [93]), education was computed as two-thirds of the adult literacy rate attained from the UNESCO Institute for Statistics 2020 [94] and one-third of the mean years of schooling obtained from the Human Development Report 2020 [95]. This score is commonly approved as representing access to education in a country [42]. Regarding family strength, the score was quantified by the ratio of divorces to marriages per 1000 members of the population consistent with previous researchers (e.g., [93]). The data was obtained from the United Nations Demographic Yearbook [96].

### 3.6. Measurement and Analysis

To perform the descriptive statistics, cross-level correlations, scale reliability, confirmatory factor analysis, convergent validity, and discriminant validity, the authors utilized SPSS software.

The framework of this study considers independent variables, dependent variables, and moderators at different levels. Thus, the authors used a hierarchical linear model (HLM) [97] to test the hypotheses. HLM was defined as a “complex form of ordinary least squares (OLS) regression that is used to analyze variance in the outcome variables when the predictor variables are at varying hierarchical levels” [98]. This technique evaluates the impacts of higher-level outcomes on lower-level ones while preserving an appropriate degree of analysis [99]. HLM has been employed in several cross-level studies (e.g., [100,101]).

## 4. Results 

Table 2 presents a matrix of correlations and sample statistics from the individual-level to country-level variables. Table 3 and Table 4 report convergent and discriminant validity test results, respectively. Finally, Table 5 illustrates results for hypotheses testing using HLM. Three models are presented in the table: those of individual-level main effects and control variables (Model 1), those of country-level main effects (Model 2), and country-level moderating effects (Model 3). 

For the confirmatory factor analysis, previous research (e.g., [102,103,104]) suggested that analysis of each variable requires at least three items. Factor analysis using statistical software will provide imprecise results if there are fewer than three items per variable [105]. Therefore, the authors only performed Confirmatory Factor Analysis (CFA) for social relatedness and work motivation.

To assess the measurement, convergent and discriminant validity were tested. Composite Reliability (CR) and Average Variance Extracted (AVE) were performed to illustrate convergent validity. The study of Hair et al. (2019) [106] suggested that CR is required to be above a threshold of 0.7. On the other hand, the AVE value should be higher than a threshold of 0.5 [107]. As shown in Table 3, CR is acceptable while AVE is slightly lower than a threshold of 0.5. Despite the limitation of AVE, the acceptable result of the discriminant validity is achieved. The discriminant validity was tested using Fornell and Larcker (1981)’s criterion [107]. This proposes that the square root of the AVE of any latent variable should be higher than its correlation with any other construct. The result of the discriminant validity test indicates that all the two latent constructs have a square root of AVE higher than its correlation with the other construct, as presented in Table 4.

The authors argued that individuals’ competence (H1), autonomy (H2), and social relatedness (H3) positively relate to their work motivation. However, the findings only supported H2 (β2 = 0.036, *p* < 0.001) and H3 (β3 = 0.042, *p* < 0.001). In contrast, the findings presented that H1 was also significant, but in the opposite direction compared with our original prediction. The result suggests that individuals’ competence negatively relates to their work motivation.

In Hypotheses 4a–d, we proposed that higher levels of religious affiliation (4a), political participation (4b), humane orientation (4c), and in-group collectivism (4d) strengthen the relationship described in H1. However, the results only demonstrated support for the two hypotheses, H4c (γ13 *=* 0.032, *p* < 0.001) and H4d (γ14 *=* 0.042, *p* < 0.001). In contrast, the findings presented that H4a was also significant, but opposite our initial prediction. This different result proposes that a higher level of religious affiliation weakens the association between individuals’ competence and work motivation.

In Hypotheses 5a–d, the authors argued that the higher levels of religious affiliation (5a), political participation (5b), humane orientation (5c), and in-group collectivism (5d) enhance the positive relationship between individuals’ autonomy and their work motivation. However, the results only supported the two hypotheses H5b (γ22 = 0.012, *p* < 0.05) and H5c (γ23 = 0.012, *p* < 0.1), while H5a and H5d were not significant.

**Figure 1:**
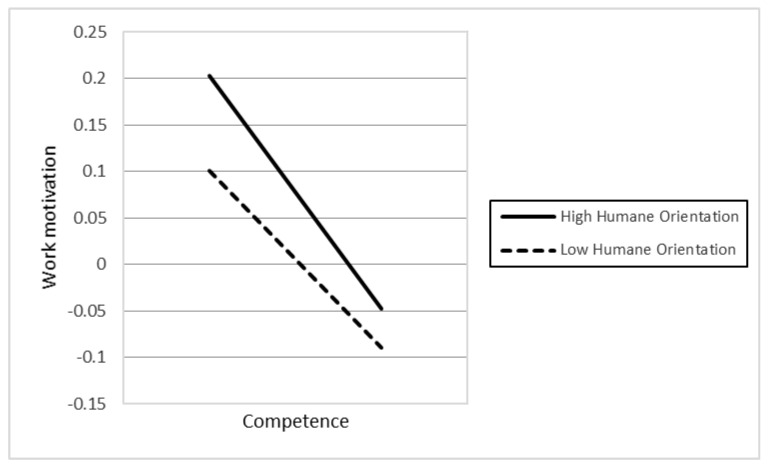
The association between competence and work motivation at different levels of humane orientation.

**Figure 2:**
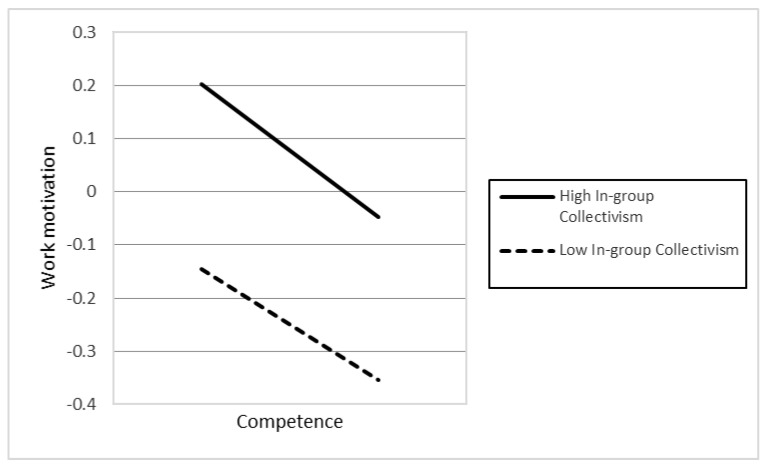
The association between competence and work motivation at different levels of in-group collectivism.

**Figure 3:**
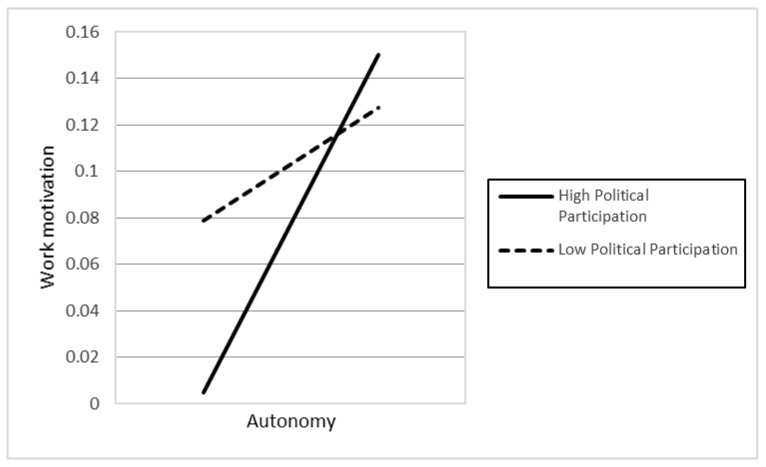
The association between autonomy and work motivation at different levels of political participation.

**Figure 4:**
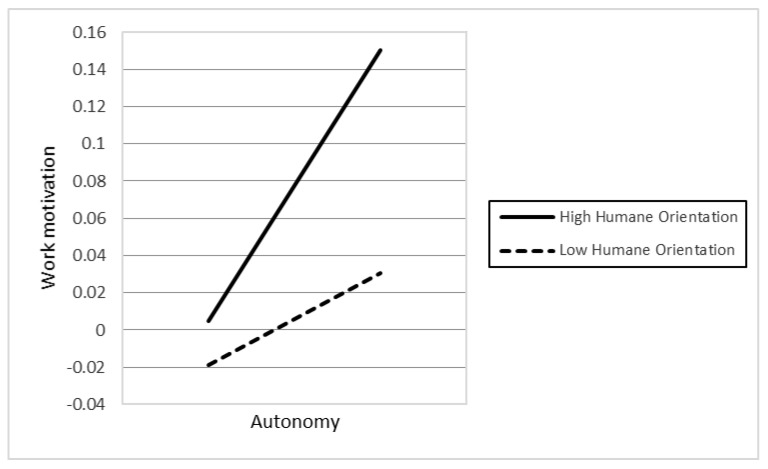
The association between autonomy and work motivation at different levels of humane orientation.

**Figure 5:**
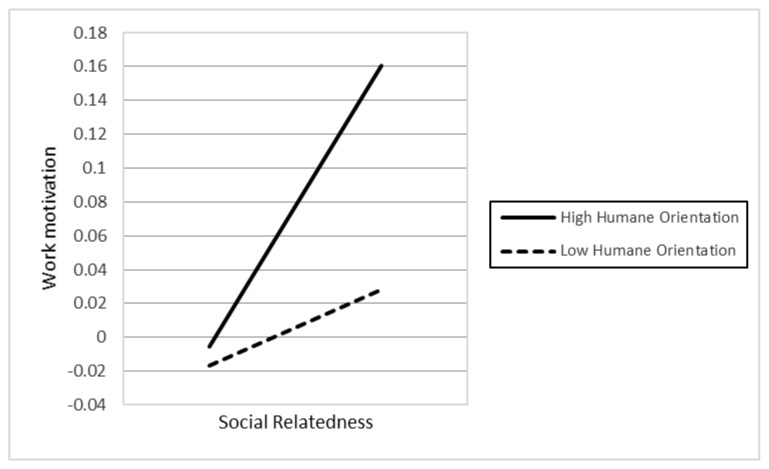
The association between social relatedness and work motivation at different levels of humane orientation.

In Hypotheses 6a–d, the authors argued that the higher levels of religious affiliation (6a), political participation (6b), humane orientation (6c), and in-group collectivism (6d) enhance the positive relationship between individuals’ social relatedness and their work motivation. However, the results only supported H6c (γ33 = 0.019, *p* < 0.01). In contrast, the findings indicated that H6d was also significant, but in the opposite direction compared to our initial hypothesis. The different result suggests that higher in-group collectivism weakens the positive association between individuals’ social relatedness and work motivation. Figure 1, Figure 2, Figure 3, Figure 4 and Figure 5 represent the significant moderators of the associations examined.

Regarding the statistical results of the control variables, gender, marital status, and age consistently indicated significant positive relationships with work motivation across three models. On the other hand, family strength indicated a significant negative association to work motivation only in Model 1.

## 5. Discussion

The study’s objective was to examine the influence of individuals’ competence, autonomy, and social relatedness on their work motivation, as well as the impact of country-level moderators, including religious affiliation, political participation, humane orientation, and in-group collectivism on their relationships. Seven primary findings are crucial in this research. First, people’s autonomy and social relatedness positively relate to their work motivation. This result is in line with the findings of prior researchers (e.g., [45,52]), postulating that humans’ autonomy and social relatedness breeds work motivation. The study of Theurer et al. (2018) [108] argued that, among motivational elements, autonomy had been found to greatly predict positive work motivation. When people feel they have enough control over their activities, they are more confident and motivated to work. Along with autonomy, humans’ social relatedness promotes communal benefits, thereby motivating people to work harder for their organization. Second, the association between individual competence and work motivation is moderated by cultural values, including humane orientation and in-group collectivism. The findings are consistent with the viewpoints of prior researchers (e.g., [69,70,77,78]), namely that a society with higher levels of humane orientation and in-group collectivism strengthens altruism, solidarity, loyalty, and the encouragement of individuals, which results in work motivation. Consequently, there will be an increase in the differences in individuals’ competence and work motivation if they live in a society with greater humane orientation and in-group collectivism. Third, political participation and humane orientation moderate the relationship between individual autonomy and work motivation. These results are in line with the investigations of prior researchers (e.g., [18,45), which found that social circumstances and cultural practices promote people’s motivation. Accordingly, the differences in individuals’ autonomy based on their work motivation will be enhanced if they belong to nations with higher political participation and humane orientation. Fourth, the association between social relatedness and work motivation is moderated by humane orientation. Accordingly, in a humane-oriented society, the differences in individuals’ social relatedness based on their work motivation will be strengthened.

The remaining findings were contrary to the original propositions. Pinder (2014) [20] argued that it is possible to find that contextual practices can influence variables at the individual level in the opposite prediction in motivation research. Fifth, individuals’ competence negatively influences their work motivation. This finding proposes that more competent individuals are less motivated at work. One possible interpretation of this opposite result is that, when the majority of the organization members recognize individuals’ competence, these individuals may perceive that it is not necessary to devote most of their time and energy to work anymore. These individuals may believe that no matter how unwillingly they perform, they are still competent enough because of their prior achievements. Additionally, competent individuals recognize that they have already sacrificed their enjoyment of life for their previous successes; therefore, they tend to offset this by investing their valuable time in other aspects. This is consistent with other researchers’ investigations (e.g., [109]), which found that low-skilled individuals are more often compelled to engage in regular work activities and are more easily motivated than others. By contrast, highly competent individuals tend to be motivated by challenging tasks and improving themselves through further education. Sixth, the relationship between competence and work motivation is negatively moderated by religious affiliation. This finding suggests that religious affiliation weakens the association between individuals’ competence and work motivation. One possible explanation for this finding is that strong religious beliefs are the foundation for virtuous living [110]. Individuals with religious affiliation usually employ religious principles to guide their behavior, regardless of their competence. In other words, both competent and incompetent individuals tend to be more motivated at the workplace if they are affiliated with any religion, thereby diminishing the influence of competence in work motivation. Seventh, the relationship between social relatedness and work motivation is negatively moderated by in-group collectivism. This result proposes that a higher degree of in-group collectivism weakens the association between individuals’ social relatedness and work motivation. One possible explanation for this is that, under an in-group collective society, people put more weight on mutual relationships and encourage acts that may build up the solidarity of groups. Since in-group collectivism is viewed as a social attachment in which people emphasize the group over the self (e.g., [79,80,81]), individuals are fairly conscious of their responsibility to the group regardless of their social relatedness. Both socially related and unrelated individuals belonging to in-group collective cultures tend to work harder for common goals. Accordingly, the influence of individuals’ social relatedness on their work motivation is reduced.

## 6. Limitations and Future Research

Despite its significant contributions, this study has its limitations. The use of secondary data represents the fact that the data collection process was beyond the authors’ control. However, the collection of cross-national data is time-consuming and costly. The authors used the available data but strove for the efficient use of multilevel data. The secondary data also limited the measurement of individual-level factors based on the available data. Moreover, it is quite complex to gauge an individual’s work motivation appropriately, since personal work motivation may not be one-dimensional. Nevertheless, the authors made efforts to employ the measurements utilized by prior research. Moreover, it is complicated to measure social factors such as political participation. There are challenges in investigating social contexts due to the absence of direct measurements [111]. This compels the authors to identify substitute measurements for this study. Finally, this study covered 25 samples from 25 countries with different characteristics. Despite the attempt of this study to include the most relevant social conditions in the framework, the influence of other national differences and cultural sensitivities were not considered.

This paper directs further research considering that several frameworks and approaches should be employed to better examine motivation [112]. First, as some of the results were opposite to the original propositions based on the theoretical foundations employed, combining different concepts and approaches is necessary to enhance perspectives of psychological needs and social issues. For instance, the relationship between competence and work motivation can be further investigated by employing other theories to understand their association better. Similarly, the moderating effects of social contexts such as religious affiliation and in-group collectivism should be further examined to obtain a more in-depth comprehension of the roles of contextual circumstances and cultural values in individual-level relationships. Additionally, self-determination theory and the concept of prosocial motivation may be used to explore motivation towards specific behavior in organizations, such as organizational citizenship and proactive behaviors. Organizational context, such as rewards, training, and culture, can be considered as part of the framework to enhance the conception of work motivation.

## 7. Conclusions

This study has utilized a multilevel framework to examine the influence of psychological needs and social context on work motivation. Through this research, a deeper understanding of the roles of competence, autonomy, and social relatedness, as well as social situations and cultural values on work motivation, is achieved. The contrary findings call for integrating other concepts and approaches towards a more comprehensive knowledge of work motivation.

Along with the theoretical contribution, the study’s findings offer practical implications. The satisfaction of psychological needs promotes self-motivation, which creates positive outcomes. Hence, organizations can provide programs and activities to promote employees’ autonomy and social relatedness as this will enhance their work motivation. Employee empowerment can be advocated by encouraging them to make their own decisions at the workplace, providing constructive criticisms rather than instilling the fear of failure. Additionally, managers should encourage solidarity, support, and mutual care among employees. Putting more weight on employees’ fulfillment of needs will further increase employees’ motivation, thereby diminishing costs related to stress or turnover [50]. To establish a novel mechanism towards promoting work motivation in the entire nation, the government should pay attention to the political structure and conditions that encourage citizens’ participation. Additionally, a culture of humane orientation should be promoted in the workplace and society so that solidarity, kind assistance, and altruism among communities as well as among individuals can be strengthened. For instance, teamwork should be encouraged for employees to help each other overcome difficulties at the workplace or share responsibilities with their colleagues. This will motivate people to work harder for collective goals, contributing to the development of organizations.

## Figures and Tables

**Table 1:** Several investigated predictors of work motivation in general and intrinsic motivation in particular.

Predictors of Work Motivation	Authors
Personal factors (age, gender, educational level, living setting, health status, and family support)	Lin, 2020 [13]
Emotional intelligence	Bechter et al., 2021 [14]
Interpersonal relationship quality	
Social exchange	Hinsz, 2008 [10]
Interaction among individuals	
Contextual factors	
Cultures	Bhagat et al., 1995 [15]; Erez, 1994/1997/2008 [16,17,18]
Social situations	Deci & Ryan, 2012 [19]
Psychological needs (but inadequacy)	Olafsen et al., 2018 [8]

**Table 2:** Descriptive statistics, cross-level correlations and scale reliability ^a,b,c^.

	Mean	SD	1	2	3	4	5	6	7	8	9	10	11	12	13
*1. Work motivation*	3.52	0.66	(0.6)												
**Individual-level variables**															
*2. Competence (β1)*	3.72	2.03	−0.160 **												
*3. Autonomy (β2)*	7.12	2.20	0.014 **	0.067 **											
*4. Social relatedness (β3)*	3.07	4.31	0.012 *	0.024 **	0.059 **	(0.9)									
**Country-level moderators**															
*5. Religious affiliation (γ01)*	83.55	18.49	0.186 **	−0.165 **	0.043 **	0.076 **									
*6. Political participation (γ02)*	66.01	18.29	−0.077 **	−0.076 **	0.081 **	0.064 **	0.215 **								
*7. Humane orientation (γ03)*	4.15	0.45	0.150 **	−0.180 **	−0.014 *	0.173 **	0.258 **	0.097 **							
*8. In-group collectivism (γ04)*	5.32	0.66	0.329 **	−0.239 **	−0.068 **	−0.057 **	0.464 **	−0.091 **	0.334 **						
**Individual-level controls**															
*9. Gender (β4)*	0.45	0.50	0.072 **	0.082 **	−0.005	−0.002	−0.016 **	−0.028 **	−0.050 **	−0.010					
*10. Marital Status (β5)*	0.57	0.50	0.036 **	−0.060 **	−0.018 **	0.014 *	−0.055 **	−0.008	0.092 **	0.021 **	0.020 **				
*11. Age (β6)*	44.17	16.34	−0.034 **	−0.186 **	−0.023 **	−0.021 **	−0.204 **	0.020 **	−0.075 **	−0.192 **	0.030 **	0.248 **			
*12. Income (β7)*	4.79	2.07	−0.046 **	0.299 **	0.136 **	0.056 **	−0.001	0.029 **	−0.034 **	−0.102 **	0.036 **	0.043 **	−0.109 **		
**Country-level controls**															
*13. Education (γ05)*	65.40	7.31	−0.035 **	0.005	−0.043 **	−0.051 **	−0.111 **	−0.069 **	−0.226 **	0.087 **	0.013 *	0.011	0.002	−0.038 **	
*14. Family strength (γ06)*	0.30	0.17	−0.227 **	0.195 **	0.015 **	−0.099 **	−0.384 **	0.017 **	−0.393 **	−0.450 **	0.040 **	−0.054 **	0.157 **	0.058 **	0.206 **

^a^ *n* = 32,614 level 1; *n* = 25, level 2. ^b^ * *p* < 0.05, ** *p* < 0.01. ^c^ The reliability found in the parentheses is expressed as Cronbach’s alpha for scales with ≥four items.

**Table 3:** Convergent validity.

	Composite Reliability (CR)	Average Variance Extracted (AVE)
Work motivation	0.744	0.431
Social relatedness	0.889	0.404

**Table 4:** Discriminant validity—Fornell and Larcker’s criterion.

	Work Motivation	Social Relatedness
Work motivation	0.657	
Social relatedness	0.012 *	0.636

* *p* < 0.05.

**Table 5:** HLM results: (The DV is work motivation) ^a,b^.

	Model 1			Model 2			Model 3		
	Coefficient	SE		Coefficient	SE		Coefficient	SE	
**Individual-level main effect**									
*Competence (β1)*	−0.063	0.006	***	−0.063	0.006	***	−0.063	0.006	***
*Autonomy (β2)*	0.036	0.005	***	0.037	0.005	***	0.036	0.005	***
*Social relatedness (β3)*	0.042	0.006	***	0.042	0.006	***	0.042	0.006	***
**Country-level main effect**									
*Religious affiliation (γ01)*				0.010	0.061		0.007	0.062	
*Political participation (γ02)*				−0.064	0.054		−0.064	0.055	
*Humane orientation (γ03)*				0.019	0.059		0.033	0.060	
*In-group collectivism (γ04)*				0.297	0.066	***	0.288	0.067	***
**Country-level moderating effect**									
*Competence x* *Religious affiliation (γ11)*							−0.013	0.007	†
*Competence x Political participation (γ12)*							−0.000	0.006	
*Competence x* *Humane orientation (γ13)*							0.032	0.007	***
*Competence x In-group collectivism (γ14)*							0.042	0.007	***
*Autonomy x* *Religious affiliation (γ21)*							−0.009	0.007	
*Autonomy x Political participation (γ22)*							0.012	0.006	*
*Autonomy x* *Humane orientation (γ23)*							0.012	0.006	†
*Autonomy x In-group collectivism (γ24)*							0.011	0.007	
*Social relatedness x Religious affiliation (γ31)*							−0.006	0.009	
*Social relatedness x Political participation (γ32)*							−0.013	0.008	
*Social relatedness x Humane orientation (γ33)*							0.019	0.007	**
*Social relatedness x In-group collectivism (γ34)*							−0.020	0.008	*
**Individual-level controls**									
*Gender (β4)*	0.067	0.005	***	0.067	0.005	***	0.068	0.005	***
*Marital Status (β5)*	0.011	0.006	*	0.011	0.005	*	0.013	0.006	*
*Age (β6)*	0.025	0.006	***	0.026	0.006	***	0.027	0.006	***
*Income (β7)*	0.002	0.006		0.002	0.006		0.003	0.006	
**Country-level controls**									
*Education (γ05)*	−0.014	0.079		−0.054	0.056		−0.052	0.057	
*Family strength (γ06)*	−0.218	0.080	*	−0.067	0.062		−0.077	0.062	

^a^, *n* = 32,614 level 1; *n* = 25, level 2. ^b^, †, *p* < 0.10, * *p* < 0.05, ** *p* < 0.01, *** *p* < 0.001.

## Data Availability

The data that support this study are publicly available.
